# Generation of hiPSCs with *ABO* c.767T>C substitution: resulting in splicing variants

**DOI:** 10.3389/fgene.2023.1141756

**Published:** 2023-06-15

**Authors:** Yinge Jin, Tao Chen, Wei Zheng, Jiahui Xi, Yin Zi, Jinling Wang, Yue Chi, Min Chen, Qingjian Zou, Chengcheng Tang, Liangxue Lai, Xiaoqing Zhou

**Affiliations:** ^1^Guangdong Provincial Key Laboratory of Large Animal Models for Biomedicine, School of Biotechnology and Health Science, South China Institute of Large Animal Models for Biomedicine, Wuyi University, Jiangmen, China; ^2^ Chinese Academy of Sciences, Guangzhou Institutes of Biomedicine and Health, Guangzhou, China

**Keywords:** base substitution, ABO gene, hiPSC, splicing variants, rare subtype

## Abstract

**Introduction:** The ABO blood group system has important clinical significance in the safety of blood transfusion and organ transplantation. Numerous ABO variations, especially variations in the splice sites, have been identified to be associated with some ABO subtypes.

**Methods:** Here, we performed the c.767T>C substitution of the *ABO* gene in human induced pluripotent stem cells (hiPSCs) by the adenosine base editor (ABE) system and described its characteristics at the genome level in detail.

**Results:** The hiPS cell line with c.767T>C substitution maintained a normal karyotype (46, XX), expressed pluripotency markers, and showed the capability to spontaneously differentiate into all three germ layers *in vivo*. The genome-wide analysis demonstrated that the c.767T>C substitution in the *ABO* gene did not cause any detected negative effect in hiPSCs at the genome level. The splicing transcript analysis revealed that splicing variants were observed in the hiPSCs with *ABO* c.767T>C substitutions.

**Conclusion:** All these results indicated that some splicing variants occurred in hiPSCs with c.767 T>C substitution of ABO gene, which probably had a significant effect on the formation of the rare ABO*Ael05/B101 subtype.

## 1 Introduction

The discovery of the ABO blood group system by Karl Landsteiner is fundamental to the safety of blood transfusion and organ transplantation, which requires the identification of weak phenotypes or subgroups (Landsteiner, 1990; Landsteiner, 1991). The system is mainly defined by the presence or absence of carbohydrate antigens on the surface of red blood cells and their corresponding antibodies in serum. As the most important element in controlling these blood group antigens, the *ABO* gene is composed of seven exons spanning approximately 20 kbp and encodes glycosyltransferases synthesizing the glycoproteins and glycolipids on the red blood cell surface and in body fluids (Yamamoto et al., 1990). Depending on the different carbohydrate antigens, the *ABO* gene can be divided into three alleles, namely, A allele, B allele, and O allele. The A and B alleles encode glycosyltransferases A (GTA) and glycosyltransferases B (GTB), respectively, while the O allele results in the absence of active glycosyltransferases (Storry and Olsson, 2009; Zhu et al., 2010). These three alleles comprise four phenotypes of the ABO blood group system, that is, A, B, O, and AB. Beyond this, some subtypes of ABO phenotypes have been identified in populations that express reduced antigens or antibodies, resulting in differences in forward and reverse ABO typing (Hosoi, 2008; Zhu et al., 2010).

Numerous known single-nucleotide polymorphisms (SNPs) and insertion- or deletion-based mutations are spread throughout the seven exons of the *ABO* gene or located in regulatory regions influencing the transferase activity (Seltsam et al., 2003; Patnaik et al., 2012). Variations in the *ABO* gene may affect the activity and specialty of glycosyltransferases, resulting in the formation of the ABO subtypes and even rare blood types (Seltsam et al., 2005; Chen et al., 2015). A rare blood type refers to the lack of common blood group antigens in human red blood cells, such as Rh, MNS, Lewis, Diego, Kell, Kidd, Duffy systems, and so on (Du et al., 2021). Because of the limited storage of blood, rare blood types, especially rare subtypes, should receive special attention in blood transfusion.

The c.767T>C substitution in exon 7 of the *ABO* gene, which resulted in an isoleucine to threonine substitution at codon 256, led to the decreased expression of A antigen and produced a new rare ABO*Ael05/B101 subtype (Feng et al., 2017). The new rare ABO*Ael05/B101 subtype was difficult to identify by routine phenotyping and showed only a few cases around the world. Such a rare blood subtype requires more attention for blood resources and blood transfusion strategies. It is necessary to study the mechanism of c.767T>C substitution in the *ABO* gene to provide clinical guidance for Ael subtype blood transfusion. In addition, whether the c.767T>C substitution in the *ABO* gene can affect at the whole genome level remains unclear and requires further research.

Given that variations, such as SNPs and indels, in the *ABO* gene can cause splicing variants, resulting in the formation of ABO subtypes, we wondered whether the *ABO* c.767T>C substitution resulted in the ABO*Ael05/B101 subtype through the splicing variant. Human induced pluripotent stem cells (hiPSCs) can differentiate into various cells, such as hematopoietic stem cells or blood cells, and can provide a good cell model to study the mechanism of the *ABO* c.767T>C substitution at different stages of cell development. In this study, we performed the c.767T>C substitution of the *ABO* gene in hiPSCs and described its characteristics at the genome and transcriptome levels. The hiPSC line with the c.767T>C substitution maintained a normal karyotype (46, XX), expressed pluripotency markers, and showed the capability to spontaneously differentiate into all three germ layers *in vivo*. The splicing transcript analysis revealed that splicing variants were observed in the hiPSCs with the *ABO* c.767T>C substitution, which probably had a significant effect on the formation of the ABO subtype.

## 2 Results

### 2.1 Design of base editing reagents to induce substitution of c.767T>C in human *ABO* gene

**FIGURE 1:**
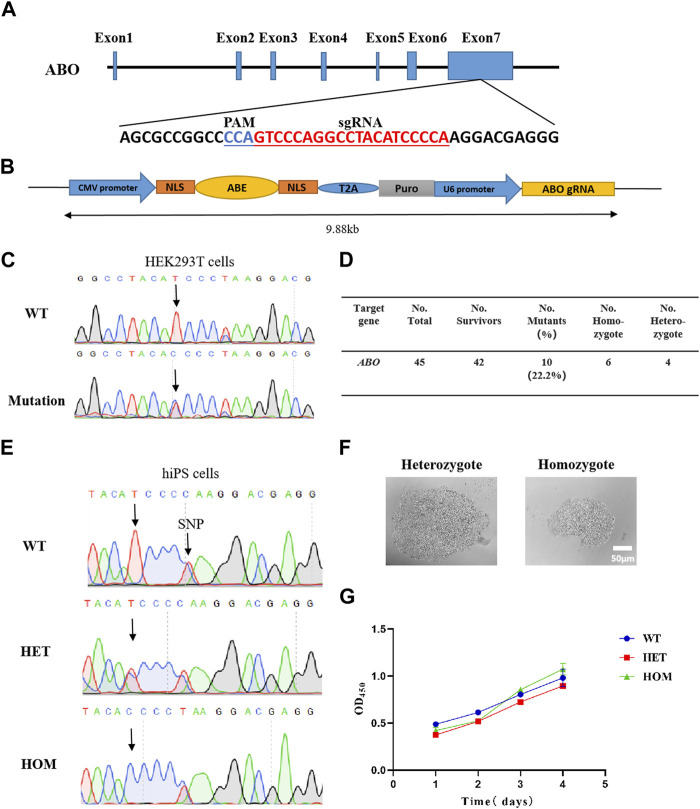
Generation of base-editing reagents and identification of the c.767T>C substitution of the human *ABO* gene in cells. **(A)** Sequences of sgRNA targeting *ABO* exon 7. **(B)** Representation of the designed plasmid. **(C)** Sanger sequencing results show *ABO* c.767T>C substitution in HEK293T cells. **(D)** Summary results of cell screening. **(E)** Sanger sequencing results show *ABO* c.767T>C substitution in hiPSCs. **(F)** Morphology of hiPSC monoclones. **(G)** Growth rate of the cells. WT, wild-type clones; HOM, homozygous clones; and HET, heterozygous clones.

The ABE editing system is a gene editing tool developed based on CRISPR/Cas9 and adenine deaminase that converts A/T into G/C in the target range (Gaudelli et al., 2017). Based on the design concept of the ABE system, we designed a small guide RNA (sgRNA) that can target an amino acid at position 256 in exon 7 of the *ABO* gene (Figure 1A). The sgRNA sequences were cloned into the ABEmax plasmid, which contained Cas9, adenosine deaminase, and the puromycin resistance gene (Figure 1B). In order to verify the targeting effect of sgRNA and editing efficiency of the ABE system, we first selected HEK293T cells for verification. We transfected the constructed plasmids into the HEK293T cells with lipofectamine and subsequently added puromycin for 3 days. The surviving cells were collected and then analyzed by sequencing PCR products covering the target locus. The results showed the predicted c.767T>C substitution at the target site, and the base editing efficiency was approximately close to 50% (Figure 1C).

### 2.2 Successfully induced c.767T>C substitution of the *ABO* gene in hiPSCs

After verifying the role of the sgRNA in HEK293T cells, we transfected the expression vector into hiPSCs by electroporation instead of lipofectamine, considering its low transfection efficiency in iPSCs. After puromycin screening for 2 weeks, a total of 45 monoclonal strains were obtained, and 42 strains survived, of which 10 were expected to have c.767T>C substitutions, that is, six homozygotes and four heterozygotes (Figure 1D). The homozygote and heterozygote all performed normal genotype and typical stem cell morphology, respectively (Figures 1E, F). Trypan blue staining showed the cell viability of the homozygous clone and heterozygous clone were similar when compared with wild-type (WT) hiPSCs (Supplementary Figure S1A). The CCK8 experiment demonstrated that there was no difference in the survival rate among the modified hiPSCs and WT hiPSCs (Figure 1G). These data demonstrated that the sgRNA we designed can achieve considerable c.767T>C substitution of the *ABO* gene in hiPSCs, with a modified rate of up to 22.2% (Figure 1D).

### 2.3 Characteristics of hiPSC clones with *ABO* c.767T>C substitution

**FIGURE 2:**
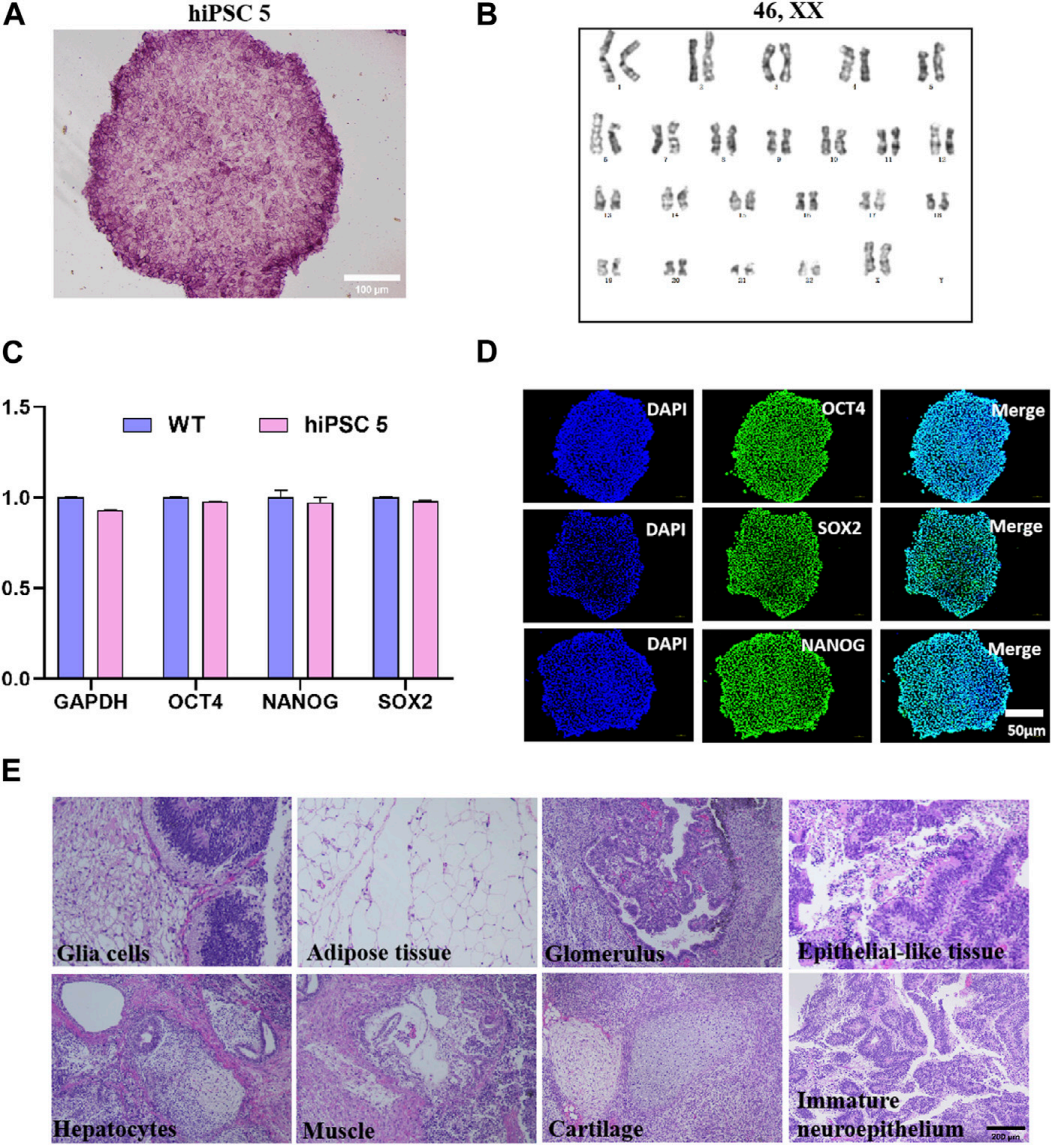
Characterization of homozygote hiPSC 5. **(A)** Typical alkaline phosphatase activities of hiPSC 5. **(B)** Karyotype of hiPSC 5. **(C)** Pluripotent gene expression levels of hiPSC 5 and WT hiPSCs. **(D)** Immunofluorescence assay for Oct4, SOX2, and NANOG. **(E)** Teratomas assay of hiPSC 5 show various tissues of three germ layer formation *in vivo*.

Through cell screening, we obtained a total of six homozygous mutant clones and selected the homozygous clone 5 (hiPSC 5) for later experiments. The cultured hiPSC 5 showed typical alkaline phosphatase activity (Figure 2A), a normal karyotype (46, XX) (Figure 2B), and no *mycoplasma* contamination (Supplementary Figure S1B). PCR detection indicated that there was no integration of foreign genes in the hiPSC 5 genome (Supplementary Figure S1C).

In addition, by quantitative RT-qPCR, the relative expression levels of the pluripotency-related genes were determined, and the results demonstrated that hiPSC 5 expressed NANOG, OCT4, SOX2, and GAPDH at a level equal to the WT hiPSCs (Figure 2C). Immunofluorescence staining analysis showed a high level of pluripotency markers such as NANOG, OCT4, SOX2, SSEA-3, and SSEA-4 expressed in this hiPSC 5 (Figure 2D; Supplementary Figure S2). Furthermore, with the teratoma formation experiment, we observed that hiPSC 5 could differentiate into various tissues of three germ layers *in vivo* (Figure 2E). All these results demonstrated that the c.767T>C substitution in the *ABO* gene did not affect the pluripotency of hiPSC 5.

### 2.4 Genome-wide and transcriptome-wide analysis of hiPSC with *ABO* c.767T>C substitution

**FIGURE 3:**
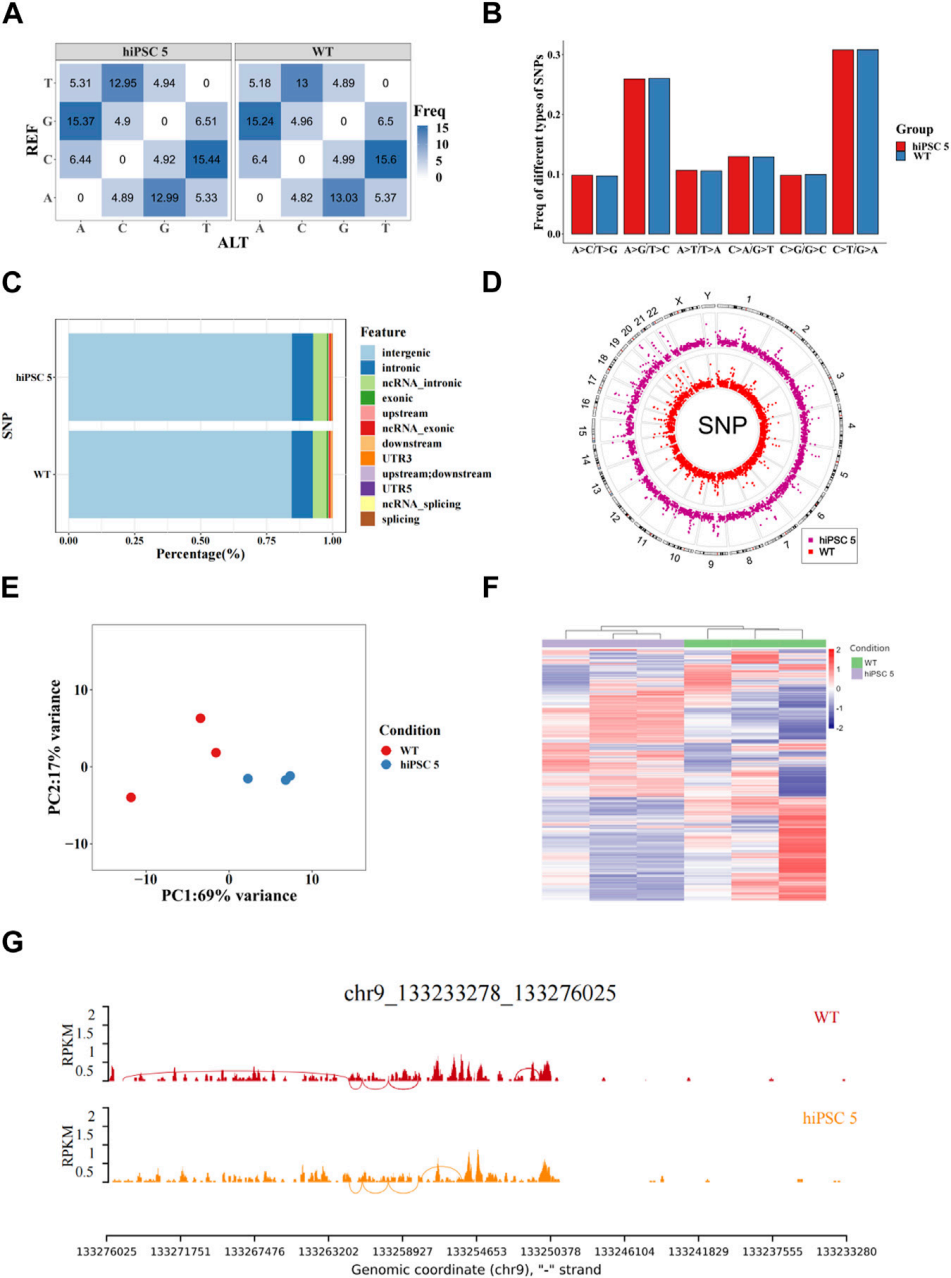
Genome-wide analysis of hiPSC 5 and WT hiPSCs. **(A)** Frequencies of indels and SNPs in hiPSC 5 and WT hiPSCs. **(B)** Frequencies of four base substitutions in hiPSC 5 and WT hiPSCs. **(C)** Peak distribution of SNPs in different genomic regions. **(D)** Distributions of SNPs on the human chromosomes in hiPSC 5 and WT hiPSCs. **(E)** Principal component analysis (PCA) of iPSC 5 and WT hiPSCs. **(F)** Heatmap of differentially expressed genes. Legend on the top right indicates the log fold change in the genes. **(G)** Alternative splicing analysis of iPSC 5 and WT hiPSCs.

To comprehensively evaluate the effect of the c.767T>C substitution of the *ABO* gene in hiPSCs, we performed whole-genome sequencing (WGS) and RNA-seq for the hiPSC 5 and WT hiPSCs. For the WGS analysis, we first aligned the sequences of the hiPSC 5 and WT hiPSCs with the reference human sequences to obtain SNPs or indels, and then the SNPs or indels in the hiPSC 5 were compared with the WT hiPSCs. For the WGS analysis, we counted the number and frequency of indels and SNPs, respectively, and the comparison showed no significant difference between the hiPSC 5 and WT hiPSCs (Figure 3A; Supplementary Figure S3A; Supplementary Table S1). Due to the characteristics of the ABE system, the SNP data were subjected to a more detailed analysis. The results indicated that the frequency of the different types of SNPs was similar between the hiPSC 5 and WT hiPSCs (Figure 3B; Supplementary Table S2). Furthermore, most SNPs in both hiPSC 5 and WT hiPSCs were in the intergenic or intronic regions (Figure 3C; Supplementary Table S3), which had less influence on gene expression, which is consistent with the reference human genome. Furthermore, the frequencies of the four base substitutions and the top 20 SNP sites were very similar, and 90% overlapped between the hiPSC 5 and WT hiPSCs (Figure 3D; Supplementary Figure S3B). All these results demonstrated that the c.767T>C substitution in the *ABO* gene did not cause any detected negative effect in hiPSC 5 at the genome level.

For the transcriptome analysis, hiPSC 5 and WT hiPSCs were performed for RNA-seq. The principal component analysis (PCA) showed that hiPSC 5 replicates were not far away from the replicates of WT hiPSCs at the mRNA expression level (Figure 3E). In addition, the gene expression profiles were slightly different between the hiPSC 5 and WT hiPSCs (Figure 3F). Alternative splicing analysis revealed that there were splicing variants in hiPSC 5 when compared to the WT hiPSCs (Figure 3G).

### 2.5 Splicing variants observed in hiPSCs with *ABO* c.767T>C substitution

The NetGene2-2.42 software was used to predict potential splicing transcripts (Jaganathan et al., 2019). A sequence analysis of the exon and intron splicing acceptor/donor sites showed that the splice sites of ABO mRNA made a difference when comparing hiPSC 5 with WT hiPSCs. New splice sites were predicted in hiPSC 5, which were labeled with the scores 0.23, 0.17, and 0.07. Other previous splice sites were labeled with the score 0.32 as predicted to be lost in hiPSC 5 (Table 1). Although the scores were not very high, they showed that these variants had a probability of affecting the splice transcripts.

**TABLE 1:** Splice probability of the variant types *in silico* analysis using NetGene2.

Splicing site		Splice probability	
		WT	hiPSC 5
Donor splice sites, direct strand	TCC​GGA​ACC​C^GTG​AGC​GGC​T	0.95	0.95
	AGC​AGA​ACG​G^GTA​AAC​TCT​G	0.36	0.36
Donor splice sites, complement strand	GCA​GGC​CCT​G^GTG​AGC​CGC​T	0.77	0.80
	ACC​CCC​CCA​G^GTA​GTA​GAA​A	0.45	0.70
	GCG​CTC​GTA​G^GTG​AAG​GCC​T	0.32	—
	CGC​ACA​CCA​G^GTA​ATC​CAC​C	0.41	0.41
Acceptor splice sites, direct strand	CTC​AGG​ACA​G^GGC​AGG​AGA​A	0.19	0.19
	GAC​AGG​GCA​G^GAG​AAC​GTG​G	0.18	0.18
	GCT​CCC​CCA​G^CCC​CCG​TCC​G	0.55	0.55
	TGC​CTT​GCA​G^ATA​CGT​GGC​T	1.00	1.00
	TTT​CCT​GAA​G^CTG​TTC​CTG​G	0.17	0.17
	CCC​ACC​ACA​G^AGA​CAC​CAT​T	0.16	0.16
Acceptor splice sites, complement strand	GCT​TCC​GTA​G^AAG​CCG​GGG​T	0.25	0.31
	CGG​GGT​GCA​G^GGT​GCC​GAA​C	0.07	0.18
	TGC​CGA​ACA​G^CGG​AGT​CAG​G	0.07	0.18
	AAC​AGC​GGA​G^TCA​GGA​TCT​C	0.07	0.17
	GCG​GAG​TCA​G^GAT​CTC​CAC​G	0.07	0.17
	GCC​CTC​CCA​G^AGC​CCC​TGG​C	0.18	0.18
	CCT​CCC​AGA​G^CCC​CTG​GCA​G	0.18	0.18
	CCC​CTG​GCA​G^CCG​CTC​ACG​G	0.19	0.19
	GGC​ACC​GCA​G^TGA​ACC​TCA​G	0.18	0.18
	TGA​ACC​TCA​G^CTT​CCT​CAG​G	0.17	0.17
	GCT​TCC​TCA​G^GAC​GGC​GGG​C	0.14	0.14
	ACT​TGT​TCA​G^GTG​GCT​CTC​G	0.17	0.17
	GCT​TCC​GTA​G^AAG​CCG​GGG​T	—	0.23
	TCC​GTA​GAA​C^CCG​GGG​TGC​A	—	0.17
	CGG​GGT​GCA​G^GGT​GCC​GAA​C	—	0.07

**FIGURE 4:**
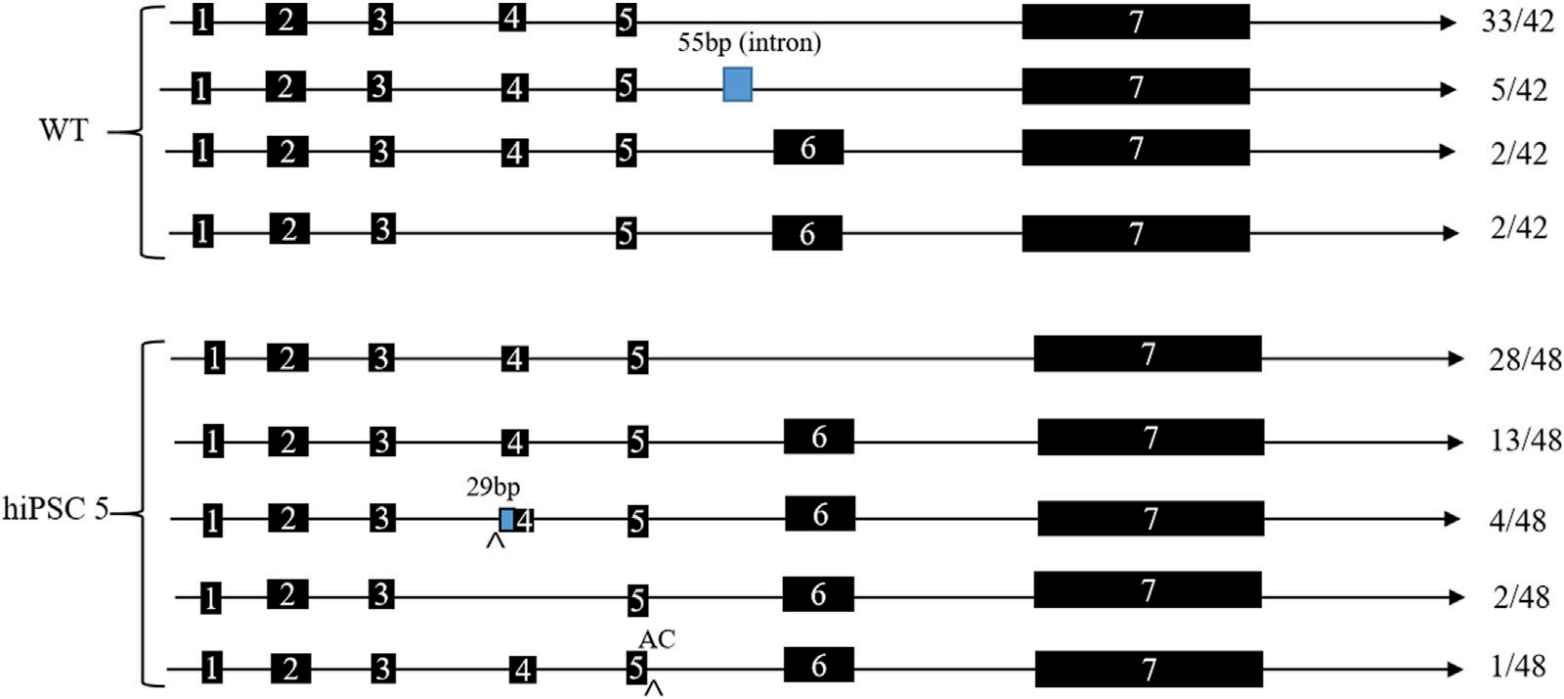
ABO mRNA splicing patterns of hiPSC 5 and WT hiPSCs. The number listed to the right is the TA clone number, which had been sequenced.

In order to verify the predicted splicing variants, we designed a forward primer in exon 1 of the *ABO* gene and a reverse primer in exon 7 of the *ABO* gene and then performed RT-PCR and TA cloning detection. The results demonstrated that the majority of ABO mRNA forms lacked exon 6, and the minority lacked exon 4 among the WT and hiPSC 5 clones. While there were two splicing variants in the hiPSC 5 clone, one type was a 29-bp insertion in exon 4, and the other type was an insertion of AC at the end of exon 5 (Figure 4).

All these results indicate that some splicing variants occurred in hiPSCs with c.767T>C substitution of the *ABO* gene, which may have a significant effect on the formation of the rare ABO*Ael05/B101 subtype.

## 3 Discussion

Blood group research has important clinical significance in safe blood transfusion, fetal and neonatal hemolytic diseases, and organ transplantation. With the unremitting efforts of scientists, an increasing number of blood types have been discovered, such as various rare blood groups (Yamamoto et al., 2012; Niwa et al., 2016; Cai et al., 2017; Matzhold et al., 2020). Blood transfusions are the main treatment options for patients with severe blood loss and are an indispensable part of modern medicine. Limited rare blood group resources, blood group compatibility, and the risk of infection pose significant challenges for patients with rare blood types. Therefore, any alternative solution is helpful for patients with rare blood groups.

The Ael subtype in the Chinese population is about one in 80,000, which is one-thousandth of the Rh-negative blood type. The new rare ABO*Ael05/B101 subtype, with the c.767T>C substitution in exon 7 of the *ABO* gene, shows a decreased expression of A antigen (Feng et al., 2017), which is difficult to identify by routine phenotyping. It is important to study the cause of such a rare blood type to provide clinical guidance for Ael subtype blood transfusions.

In our study, we performed the c.767T>C substitution of the *ABO* gene in hiPSCs by the ABE system and described its characteristics at the genome level in detail. The WGS analysis demonstrated that the c.767T>C substitution in the *ABO* gene did not cause any detected negative effect in hiPSC 5 at the genome level. *In silico* analysis revealed that the c.767T>C substitution caused splicing variants with a low score of 0.07–0.32. RT-PCR and TA clone detection showed that the majority of ABO mRNA forms lacked exon 6, which is consistent with other research works (Chen et al., 2015). While there were two different splicing variants in the homozygote, one pattern was a 29-bp insertion in exon 4 while another was an insertion of AC at the end of exon 5. We speculated that these splicing variants probably had a significant effect on the formation of the rare ABO*Ael05/B101 subtype. Many reports have verified that variations in the *ABO* gene, such as SNPs, resulted in the formation of ABO subtypes (Seltsam et al., 2003; Seltsam et al., 2005; Patnaik et al., 2012; Chen et al., 2015).

The hiPSCs with the *ABO* c.767T>C substitution maintained pluripotency and could spontaneously differentiate into all three germ layers, which could provide a good cell model for the research of the rare ABO subtype in the different stages of cell development. Once these modified hiPSCs were induced to differentiate into hematopoietic stem cells, even different blood cells, we could study the exact mechanism of the ABO subtype more comprehensively to broaden the rare blood resources. Some methods of hematopoietic differentiation can make hiPSCs undergo directed differentiation into hematopoietic progenitors or blood cells (Kessel et al., 2017; Bernecker et al., 2019; Ebrahimi et al., 2020; Sivalingam et al., 2021), but efficiency and purity present significant challenges. Next, we will cooperate with an experienced group to induce the modified hiPSCs into hematopoietic stem cells or blood cells and then study the exact mechanism of the ABO subtype more comprehensively. Because the A antigen expresses on the membranes of blood cells, once hematopoietic stem cells or blood cells are induced, a Western blot can be performed to identify whether there has been any decrease in the expression of the A antigen on induced blood cells, and RNA-seq can be performed to test the whole transcriptome that include splicing variants. So, further research studies are required in the future to verify the actual cause of the ABO*Ael05/B101 subtype.

## 4 Materials and methods

### 4.1 Cell culture

The HEK293T cells were cultured with 90% DMEM and 10% FBS and incubated at 37°C in humidified air with 5% CO_2_. The hiPSCs were derived from the umbilical cord blood CD34^+^ cells, which were reprogramed by transfecting with pEP4-EO2S-ET2K, pCEP4-M2L, and pCEP4-miR302 clusters. All hiPSCs were cultured on 1% Matrigel-coated plates in an mTeSR1 medium (stem cell) and incubated at 37°C in humidified air with 5% CO_2_. The cells were passaged after reaching approximately 80% confluence.

### 4.2 CRISPR/Cas9-mediated gene editing

The sgRNA targeting the sequence GTC​CCA​GGC​CTA​CAT​CCC​CA of exon 7 of the *ABO* gene was designed and then combined with ABEmax and puromycin resistance genes to form a new expression plasmid. The expression plasmid was transfected into HEK293T cells with Lipofectamine 8000. After 24 h of recovery, the cells were screened with 1,000 ng/mL of puromycin for 3 days, and the surviving cells were collected for PCR and Sanger sequencing. For hiPSCs, we transfected the expression plasmid by electroporation (Pulse Generator CUY21EDIT Ⅱ). After 24 h of culture, the electroporated hiPSCs were selected with 500 ng/mL puromycin for 2 weeks. Individual cell colonies were picked up and cultured into 96-well plates, and a portion of each clone was subjected to PCR amplification and DNA sequencing.

### 4.3 Genomic DNA extraction and Sanger sequencing

Single clones were collected, and the genomic DNA was extracted using the TIANamp Genomic DNA Kit (TIANGEN, #DP304-03). The fragments containing the target site were amplified by PCR (forward primer, ACC​ACG​TGG​GCG​TGG​AGA​T; reverse primer, AGA​GCA​CCT​TGG​TGG​GTT​TG) and sent to Tsingke Biotechnology Co., Ltd., for Sanger sequencing. The sequencing results were analyzed by using SnapGene.

### 4.4 *Mycoplasma* test

All hiPSCs were resuscitated and cultured normally for 2 days, and then the culture medium was collected for PCR (Vazyme MycoBlue Mycoplasma Detector, Vazyme, D101-02).

### 4.5 Karyotype analysis

A total of 1 × 10^6^ hiPSCs were treated with colcemid for 130 min at 37°C. Then, all cells were lysed in a hypotonic solution. Chromosomes were identified according to the International System for Human Cytogenetic Nomenclature using the standard G-banding technique.

### 4.6 RNA isolation and real-time quantitative PCR

A total RNA extraction kit (Solarbio, #1200) was used to extract total RNA according to the manufacturer’s instructions. The First Strand cDNA Synthesis Kit (EZBioscience, KR0501-100) was used to obtain cDNA. Then, 100 ng of cDNA was used for real-time quantitative PCR (RT-qPCR) reactions with 2× SYBR Green qPCR Master Mix (Bimake, #B21202) by LightCycler^®^ 96 System (Roche). The primers are listed in Supplementary Table S4.

### 4.7 Alkaline phosphatase staining

All hiPSCs were fixed using a fixative solution and stained using a dye working solution for about 22 min. The dye working solution was prepared from solution A:solution B:solution C according to 1:1:1. All reagents were from the Alkaline Phosphatase Staining Kit (BestBio, BB-44275).

### 4.8 Immunofluorescence staining

The hiPSCs were immobilized with 4% paraformaldehyde (PFA) at room temperature for 15 min. Next, all cells were permeated with 0.5% Triton X-100 for 20 min and subsequently blocked with 3% BSA for 30 min at room temperature. Then, the cells were incubated overnight at 4°C with primary antibodies (Oct4, Abcam #ab181557; SOX2, Abcam #ab137385; Nanog, Abcam #ab109250; SSEA-3, eBioscience #12-8843; and SSEA-4, BioLegend #330320). The samples were washed thrice with PBS and incubated with secondary antibodies Alexa Fluor^®^ 488 (Abcam, #ab150077) for 1 h at room temperature. The nuclei were stained with DAPI (Beyotime, #C1002). All pictures were taken with an inverted fluorescence microscope (Nikon ECLIPSE Ti2).

### 4.9 Teratoma formation assay

A total of 1 × 10^6^ cells per site were injected subcutaneously into two 8-weeks-old SCID mice. After 3 weeks, teratomas appeared at the injected sites. Then, all teratomas were collected, sectioned, and stained with hematoxylin and eosin (H&E) according to the manufacturer’s instructions.

### 4.10 Trypan blue staining

The WT hiPSC clones, homozygous clones, and heterozygous clones were re-suspended in 12-well plates with 1% Matrigel and digested into cell suspensions after 3 days of culture. Next, all cell suspensions were mixed evenly with 0.4% trypan blue solution (GenXion) at a ratio of 9:1, and the cells were visualized under a microscope. Viable cells were colorless, and dead cells were blue.

### 4.11 Cell proliferation experiment

The WT hiPSC clones, homozygous clones, and heterozygous clones were re-suspended in 6-well plates with 1% Matrigel, and when the density reached 70%, the cells were treated as a cell suspension and counted, then plated into 96-well plates with 1% Matrigel according to 4,000 cells per well in four parallel experiments for each sample. At 2, 24, 48, 72, and 96 h after passage, a mixture of an mTeSR1 medium (100 μL) and Cell Counting Kit-8 (10 μL) (Dojindo) was added. The plates were incubated in a cell culture incubator for 3 h, and the absorbance value (OD) at 450 nm was measured using a multi-mode microplate reader (BioTek Synergy Neo2).

### 4.12 Whole-genome sequencing and data analysis

For each cell sample, more than 200 μg of genomic DNA was randomly fragmented by using Covaris M220 Focused-Ultrasonicator to an average size of 300–350 bp. The fragments were end-repaired, 5′ phosphorylated, and 3′ adenylated by the End Prep Enzyme Mix to add adapters to both ends. Each cell sample was then amplified with P5 and P7 primers carrying particular sequences. The PCR products were purified into a final qualified library. The qualified libraries were pair-end sequenced at 150 bp on the Illumina HiSeq X Ten System. The data analysis was conducted as previously published (Liang et al., 2022).

### 4.13 Transcriptome sequencing and data analysis

Up to 1 μg of total RNA was used for subsequent library preparation. Isolation and fragmentation of mRNAs were performed by using Oligo (dT) beads, divalent cations, and high temperatures. The first-strand cDNA and second-strand cDNA were synthesized using random primers by reverse transcription. The purified double-stranded cDNA was then dA-tailed in one reaction and adapters were added to both ends. Size selection of the adapter-ligated DNA was performed using DNA Clean Beads. Each sample was amplified by PCR using P5 and P7 primers, and the PCR products were validated. Then, libraries with different indices were multiplexed and loaded on an Illumina HiSeq instrument for sequencing, using a 2 × 150 paired-end (PE) configuration according to the manufacturer’s instructions. The raw data were filtered and trimmed using fastp (v0.20.1) with the base quality value ≥15 (-q 15). Subsequently, Salmon (version 1.8.0) was used to align clean reads to the hg38 human transcriptome downloaded from the NCBI. In the differential analysis part, two types of hiPSCs, hiPSC 5 and WT hiPSCs, were used as samples for differential analysis, and then, the intersections of their differential genes were taken as the true differential genes. Differential expression analysis was performed using DESeq2 (version 1.20.0), and genes with an adjusted p < 0.05 and fold change >2 were considered differentially expressed.

### 4.14 Reverse transcription-PCR and TA cloning detection

The Total RNA Extraction Kit (Solarbio, #R1200) was used to extract total RNA according to the manufacturer’s instructions. Up to 1 μg of total RNA was used to perform reverse transcription using the First Strand cDNA Synthesis Kit (EZBioscience, #KR0501-100). Then, 1 μg of cDNA was used to perform PCR reactions with Ex Taq Version 2.0 (TaKaRa, #RR003) by a gene amplifier (Biometra TAdvanced) (forward primer, CCG​AGG​TGT​TGC​GGA​CGC​TG; reverse primer, TTG​GCC​TGG​TCG​ACC​ATC-ATG). Then, the reaction products were used to perform T-vector ligation using the pMDTM 19-T Vector Cloning Kit (TaKaRa, #6013).

## Data Availability

The original contributions presented in the study are publicly available. This data can be found here https://www.ncbi.nlm.nih.gov/bioproject/?term=PRJNA905801.

## References

[B1] Bernecker C. M. Ackermann N. Lachmann L. Rohrhofer H. Zaehres H. Araúzo-Bravo M. J. (2019). Enhanced *ex vivo* generation of erythroid cells from human induced pluripotent stem cells in a simplified cell culture system with low cytokine support. Stem Cells Dev. 28, 1540–1551. 10.1089/scd.2019.0132 31595840 PMC6882453

[B2] Cai X. C. Qian W. Wu H. Lei Q. Ding Q. Zou W. (2017). An exonic missense mutation c.28G>A is associated with weak B blood group by affecting RNA splicing of the ABO gene. Transfusion 57, 2140–2149. 10.1111/trf.14209 28653406

[B3] Chen D. P. Sun C. F. Ning H. C. Peng C. T. Wang W. T. Tseng C. P. (2015). Genetic and mechanistic evaluation for the weak A phenotype in Ael blood type with IVS6 + 5G>A ABO gene mutation. Vox Sang. 108, 64–71. 10.1111/vox.12196 25234298

[B4] Du F. C. Hong W. X. Wu F. Liu Y. Z. (2021). Research advances in rare blood group banks and platelet donor databases. Laboratory Med. Clin. 18, 276–278. 10.3969/j.issn.1672-9455.2021.02.041

[B5] Ebrahimi M. Forouzesh S. Raoufi M. Ramazii F. Ghaedrahmati F. Farzaneh M. (2020). Differentiation of human induced pluripotent stem cells into erythroid cells. Stem Cell Res. Ther. 11, 483. 10.1186/s13287-020-01998-9 33198819 PMC7667818

[B6] Feng Q. Xiao J. Y. Wu M. H. Zhang Y. X. Xia Y. Mu X. (2017). Identification of a rare Ael05/B101 subtype and selection of blood transfusion strategy. Zhongguo Shi Yan Xue Ye Xue Za Zhi 25, 1528–1531. 10.7534/j.issn.1009-2137.2017.05.044 29070138

[B7] Gaudelli N. M. Komor A. C. Rees H. A. Packer M. S. Badran A. H. Bryson D. I. (2017). Programmable base editing of A•T to G•C in genomic DNA without DNA cleavage. Nature 551, 464–471. 10.1038/nature24644 29160308 PMC5726555

[B8] Hosoi E. (2008). Biological and clinical aspects of ABO blood group system. J. Med. Invest. 55, 174–182. 10.2152/jmi.55.174 18797129

[B9] Jaganathan K. Kyriazopoulou P. S. McRae J. F. Darbandi S. F. Knowles D. Li Y. I. (2019). Predicting splicing from primary sequence with deep learning. Cell 176, 535–548. 10.1016/j.cell.2018.12.015 30661751

[B10] Kessel K. U. Bluemke A. Schöler H. R. Zaehres H. Schlenke P. Dorn I. (2017). Emergence of CD43-expressing hematopoietic progenitors from human induced pluripotent stem cells. Transfus. Med. Hemother 44, 143–150. 10.1159/000477357 28626365 PMC5473062

[B11] Landsteiner K. (1991). Ueber agglutinationserscheinungen normalen menschlichen blutes. Australia: Wien Klin Woschenschr, 1132–1134. 11732110

[B12] Landsteiner K. (1990). Zur Kenntnis der antifermentativen, lytischen und agglutinierenden Wirkungen des Blutserums und der Lymphe. Zentralblatt Bakteriol. 1990, 357–362.

[B13] Liang Y. Xie J. Zhang Q. Wang X. Gou S. Lin L. (2022). Agbe: A dual deaminase-mediated base editor by fusing CGBE with ABE for creating a saturated mutant population with multiple editing patterns. Nucleic Acids Res. 50, 5384–5399. 10.1093/nar/gkac353 35544322 PMC9122597

[B14] Matzhold E. M. Drexler C. Wagner A. Bernecker C. Pessentheiner A. Bogner-Strauß J. G. (2020). A 24-base pair deletion in the ABO gene causes a hereditary splice site defect: A novel mechanism underlying ABO blood group O. Transfusion 60, 1564–1572. 10.1111/trf.15907 32500601 PMC7496400

[B16] Niwa R. Nakayama T. Ishii H. Fujita E. Ishiyama K. Matsuo T. (2016). Identification of a novel missense mutation (563G>a) in the ABO gene associated with a Bel phenotype. Transfusion 56, 1242–1243. 10.1111/trf.13507 26841695

[B17] Patnaik S. K. Helmberg W. Blumenfeld O. O. (2012). Bgmut: NCBI dbRBC database of allelic variations of genes encoding antigens of blood group systems. Nucleic Acids Res. 40, D1023–D1029. 10.1093/nar/gkr958 22084196 PMC3245102

[B18] Seltsam A. Das G. C. Wagner F. F. Blasczyk R. (2005). Nondeletional ABO*O alleles express weak blood group A phenotypes. Transfusion 45, 359–365. 10.1111/j.1537-2995.2005.04228.x 15752153

[B19] Seltsam A. Hallensleben M. Kollmann A. Burkhart J. Blasczyk R. (2003). Systematic analysis of the ABO gene diversity within exons 6 and 7 by PCR screening reveals new ABO alleles. Transfusion 43, 428–439. 10.1046/j.1537-2995.2003.00321.x 12662274

[B20] Sivalingam J. Su E. Y. Lim Z. R. Lee A. P. Lim H. L. Chen H. Y. (2021). A scalable suspension platform for generating high-density cultures of universal red blood cells from human induced pluripotent stem cells. Stem Cell Rep. 16, 182–197. 10.1016/j.stemcr.2020.11.008 PMC7897557 33306988

[B21] Storry J. R. Olsson M. L. (2009). The ABO blood group system revisited: A review and update. Immunohematology 25, 48–59. 10.21307/immunohematology-2019-231 19927620

[B22] Yamamoto F. Cid E. Yamamoto M. Blancher A. (2012). ABO research in the modern era of genomics. Transfus. Med. Rev. 26, 103–118. 10.1016/j.tmrv.2011.08.002 21945157

[B23] Yamamoto F. Clausen H. White T. Marken J. Hakomori S. (1990). Molecular genetic basis of the histo-blood group ABO system. Nature 345, 229–233. 10.1038/345229a0 2333095

[B24] Zhu F. Tao S. Xu X. Ying Y. Hong X. Zhu H. (2010). Distribution of ABO blood group allele and identification of three novel alleles in the Chinese Han population. Vox Sang. 98, 554–559. 10.1111/j.1423-0410.2009.01291.x 20003128

