# Supplementary material for: Generation of hiPSCs with ABO c.767T>C substitution: resulting in splicing variants
Source: Front Genet. 2023 Jun 15;14:1141756. doi: 10.3389/fgene.2023.1141756 (PMC10310534; doi:10.3389/fgene.2023.1141756)
Supplement: Supplementary file 1 [file Table1.DOCX]

**Supplementary Table S1: The number of SNPs and indels detected in hiPSC 5 and WT hiPSCs**

| **Group** | **Samples** | **Type** | **SNV_numbers** |
| --- | --- | --- | --- |
| hiPSC5 | hiPSC5 | indel | 8986 |
| WT | WT | indel | 9171 |
| hiPSC5 | hiPSC5 | snp | 76926 |
| WT | WT | snp | 76012 |

**Supplementary Table S2: The frequency of all types of SNPs in hiPSC 5 and WT hiPSCs**

| **Type** | **Group** | **Samples** | **SNP_numbers** | **Frequence** |
| --- | --- | --- | --- | --- |
| A>C/T>G | WT | WT | 7286 | 0.097131126 |
| C>G/G>C | WT | WT | 7465 | 0.099517411 |
| A>T/T>A | WT | WT | 7914 | 0.10550312 |
| C>A/G>T | WT | WT | 9678 | 0.129019357 |
| A>G/T>C | WT | WT | 19529 | 0.260345011 |
| C>T/G>A | WT | WT | 23140 | 0.308483976 |
| C>G/G>C | hiPSC5 | hiPSC5 | 7555 | 0.098211268 |
| A>C/T>G | hiPSC5 | hiPSC5 | 7561 | 0.098289265 |
| A>T/T>A | hiPSC5 | hiPSC5 | 8191 | 0.106478954 |
| C>A/G>T | hiPSC5 | hiPSC5 | 9962 | 0.129501079 |
| A>G/T>C | hiPSC5 | hiPSC5 | 19959 | 0.259457141 |
| C>T/G>A | hiPSC5 | hiPSC5 | 23698 | 0.308062294 |

**Supplementary Table S3: The distribution of SNP detected in hiPSC 5 and WT hiPSCs**

| **Group** | **Sample_name** | **Feature** | **Percentage** |
| --- | --- | --- | --- |
| WT | WT | intergenic | 74.61563624 |
| WT | WT | intronic | 16.33409661 |
| WT | WT | ncRNA_intronic | 5.877221677 |
| WT | WT | UTR3 | 0.752371606 |
| WT | WT | downstream | 0.676044052 |
| WT | WT | upstream | 0.654236179 |
| WT | WT | ncRNA_exonic | 0.512485007 |
| WT | WT | exonic | 0.250790535 |
| WT | WT | UTR5 | 0.174462981 |
| WT | WT | upstream;downstream | 0.130847236 |
| WT | WT | exonic;splicing | 0.010903936 |
| WT | WT | splicing | 0.010903936 |
| hiPSC5 | hiPSC5 | intergenic | 76.01825061 |
| hiPSC5 | hiPSC5 | intronic | 14.98998442 |
| hiPSC5 | hiPSC5 | ncRNA_intronic | 5.809036279 |
| hiPSC5 | hiPSC5 | UTR3 | 0.734475851 |
| hiPSC5 | hiPSC5 | downstream | 0.723347429 |
| hiPSC5 | hiPSC5 | upstream | 0.612063209 |
| hiPSC5 | hiPSC5 | ncRNA_exonic | 0.545292677 |
| hiPSC5 | hiPSC5 | exonic | 0.211440018 |
| hiPSC5 | hiPSC5 | upstream;downstream | 0.200311596 |
| hiPSC5 | hiPSC5 | UTR5 | 0.144669486 |
| hiPSC5 | hiPSC5 | splicing | 0.011128422 |

**Supplementary Table S4: The sequences of all primer used in RT-qPCR**

| **Genes** | **Primer sequence** |
| --- | --- |
| GAPDH | F:GGAGCGAGATCCCTCCAAAAT |
|  | R:GGCTGTTGTCATACTTCTCATGG |
| SOX2 | F:GCTACAGCATGATGCAGGACCA |
|  | R:TCTGCGAGCTGGTCATGGAGTT |
| NANOG | F:GATGCAAGAACTCTCCAACATC |
|  | R:CTGGTGGTAGGAAGAGTAAAGC |
| Oct4 | F:CGACCATCTGCCGCTTTG |
|  | R:GCCGCAGCTTACACATGTTCT |
