# Supplementary figures and images for: Generation of hiPSCs with ABO c.767T>C substitution: resulting in splicing variants
Source: Front Genet. 2023 Jun 15;14:1141756. doi: 10.3389/fgene.2023.1141756 (PMC10310534; doi:10.3389/fgene.2023.1141756)

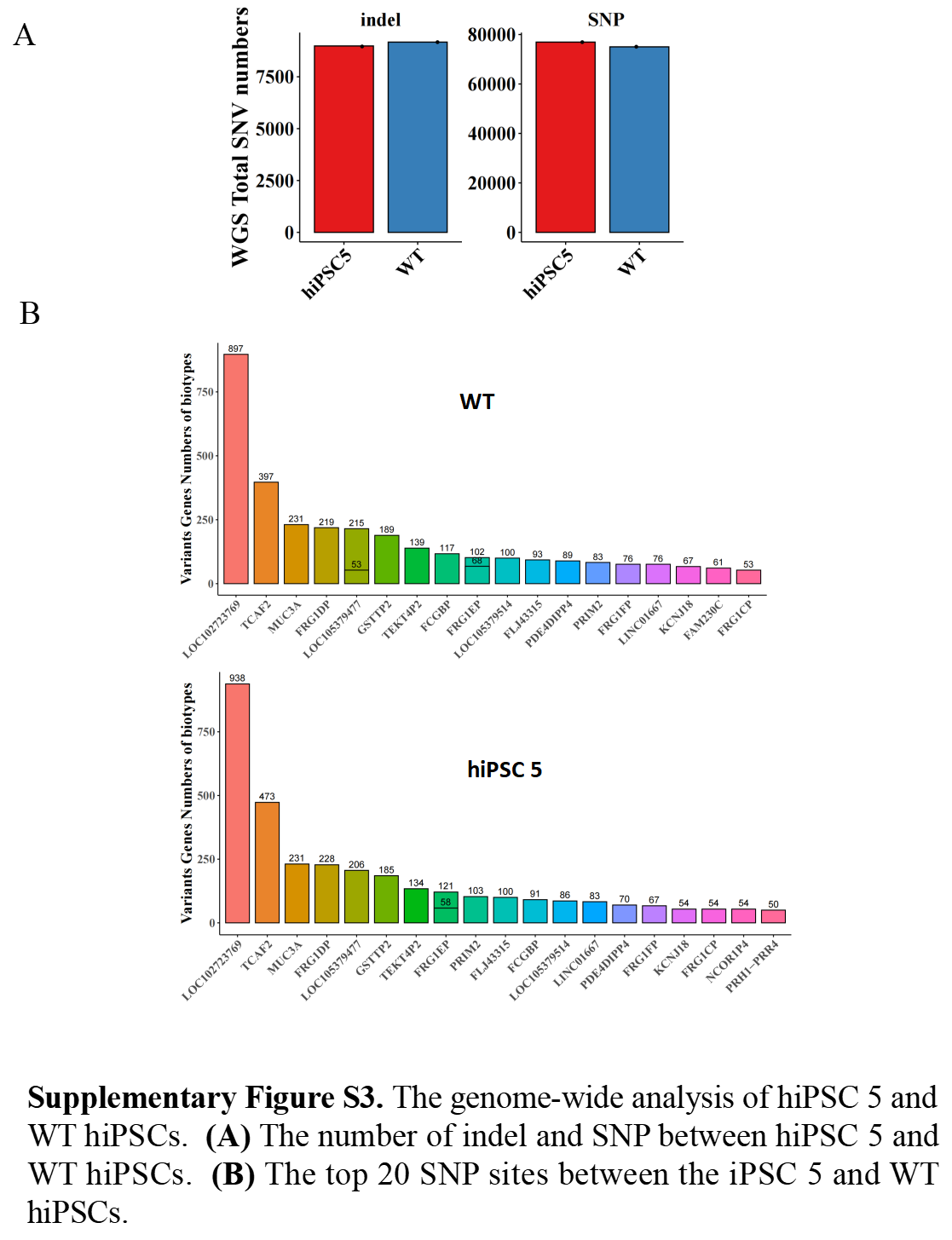

Supplement: Supplementary file 2 [file Image3.TIF]

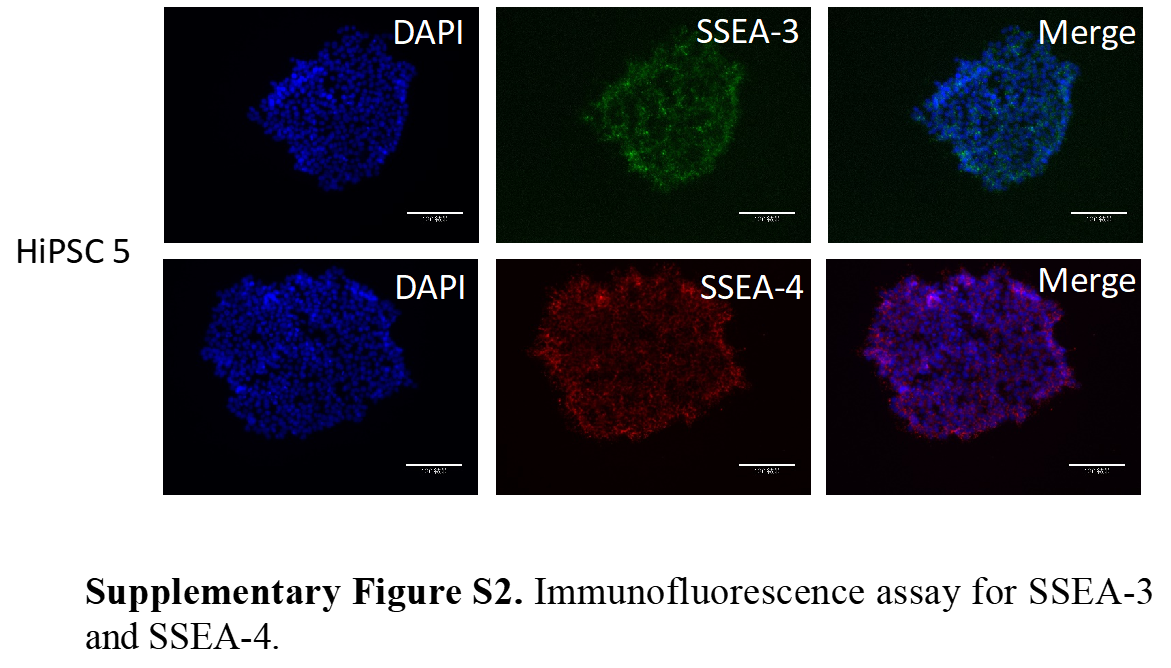

Supplement: Supplementary file 3 [file Image2.TIF]

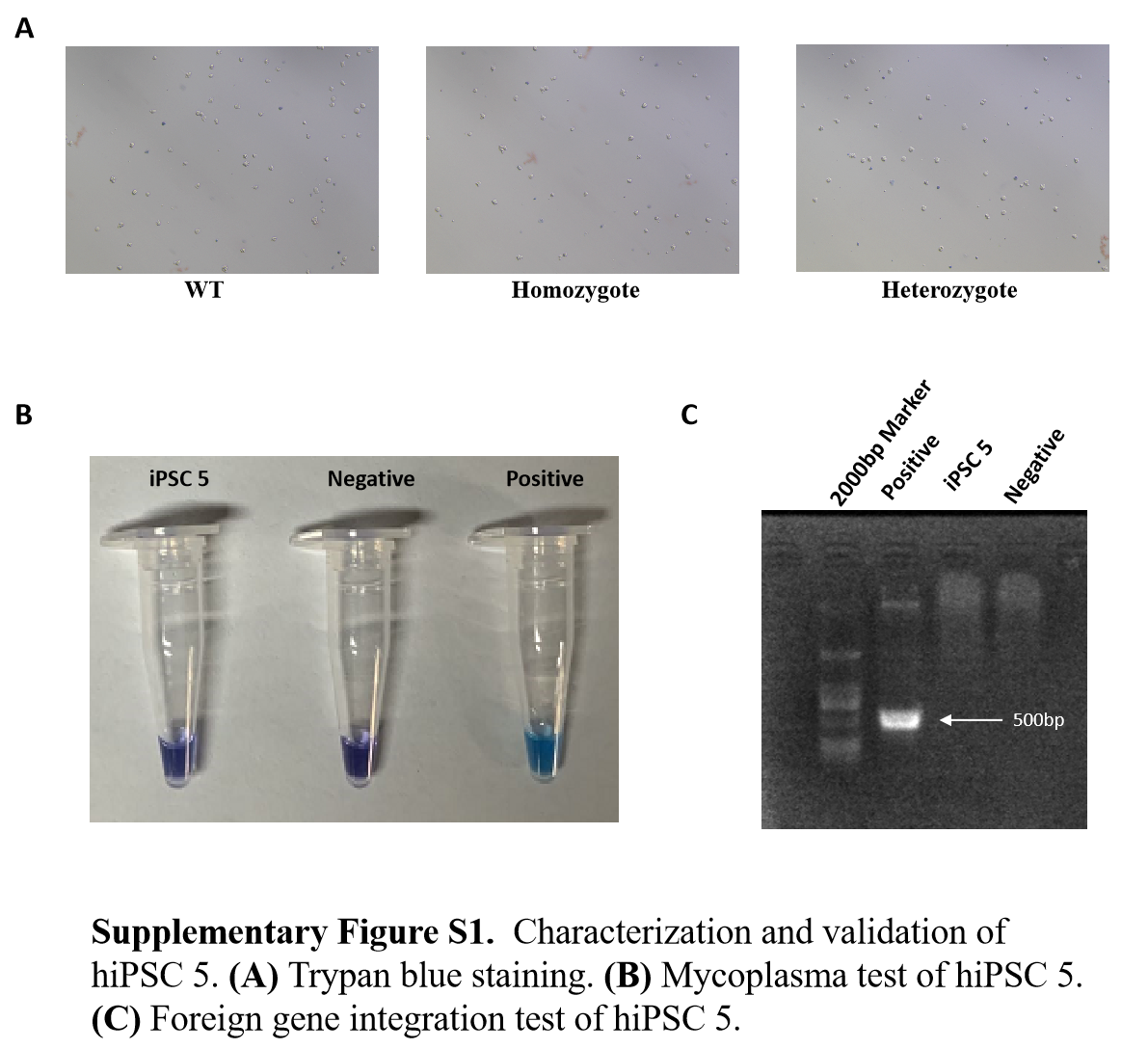

Supplement: Supplementary file 4 [file Image1.TIF]
